# Ascophyllan functions as an adjuvant to promote anti-cancer effect by dendritic cell activation

**DOI:** 10.18632/oncotarget.8200

**Published:** 2016-03-19

**Authors:** Wei Zhang, Takasi Okimura, Li Xu, Lijun Zhang, Tatsuya Oda, Minseok Kwak, Qing Yu, Jun-O Jin

**Affiliations:** ^1^ Shanghai Public Health Clinical Center, Shanghai Medical College, Fudan University, Shanghai, China; ^2^ Research and Development Division, Hayashikane Sangyo Co., Ltd., Shimonoseki, Yamaguchi, Japan; ^3^ Graduate School of Science and Technology, Nagasaki University, Nagasaki, Japan; ^4^ Department of Chemistry, Pukyong National University, Busan, South Korea; ^5^ Department of Immunology and Infectious Diseases, The Forsyth Institute, Cambridge, MA, USA; ^6^ Department of Oral Medicine, Infection and Immunity, Harvard School of Dental Medicine, Boston, MA, USA

**Keywords:** ascophyllan, adjuvant, dendritic cell maturation, cytotoxic lymphocyte activation, anti-cancer, Immunology and Microbiology Section, Immune response, Immunity

## Abstract

Our previous study demonstrated that ascophyllan, a sulfated polysaccharide purified from brown alga, has immune-activating effects. In this study, we evaluated ascophyllan as an adjuvant for its therapeutic and preventive effect on tumor in a mouse melanoma model. Ascophyllan induced migration of DCs to spleen and tumor-draining lymph node (drLN) in a mouse B16 melanoma model. Moreover, ascophyllan induced activation of dendritic cells (DCs), and promoted IFN-γ- and TNF-α-producing Th1 immune responses in tumor-bearing mice. In addition, treatment with a combination of ascophyllan and ovalbumin (OVA) in the tumor-bearing mice promoted proliferation of OVA-specific CD4 and CD8 T cells and migration of those cells into the tumor, consequently inhibiting the tumor growth. Immunization with the combination of ascophyllan and OVA caused enhanced OVA-specific antibody production and memory T cell responses compared to OVA immunization alone, and almost completely prevented B16-OVA tumor growth upon subsequent tumor challenge. Finally, the combination of ascophyllan and OVA prevented B16-OVA tumor invasion and metastasis into the liver. Thus, these results demonstrate that ascophyllan can function as an adjuvant to induce DC activation, antigen specific CTL activation, Th1 immune response and antibody production, and hence may be useful as a therapeutic and preventive tumor vaccine.

## INTRODUCTION

Tumor immunotherapy aims to induce immune responses that leads to the killing of tumor cells [1]. To achieve efficient tumor cell killing, different strategies have been evaluated for inducing T and B cell responses, especially cytotoxic T lymphocyte (CTL) activation and antigen (Ag) specific antibody production against tumor Ags [2]. However, tumor Ags, including purified and recombinant subunit of tumor Ags, are usually poorly immunogenic for T and B cell activation, because Ag presenting cells (APCs), such as dendritic cells (DCs) and macrophages, present low levels of tumor antigens through MHC molecules and express low levels of co-stimulatory molecules [3, 4]. Therefore, to enhance tumor immunity, adjuvant components with capability of inducing DC maturation, are required together with tumor Ags to promote T and B cell activation [2]. Moreover, an effective therapeutic agent against tumor needs to have immune-activating effect in the tumor environment, which is often immune-suppressive [5].

DCs are key modulators of T cell and B cell immunities [2, 6]. Immature DCs have a specific function, which in to phagocytose Ags. After exposure to stimuli or Ags, immature DCs undergo a maturation process during which they migrate to spleen and lymph nodes and induce T cell activation [2, 6]. Matured DCs produce pro-inflammatory cytokines that determine the induction of specific types of effector T cells and CTLs [7, 8]. Different mouse DCs subsets have distinct abilities and modes of Ag-presentation and T cell activation. CD8α^+^ conventional DCs (cDCs), a minor population among total mouse spleen DCs, have the selective ability to cross-present endogenously synthesized proteins, such as viral infection-expressed proteins and certain exogenous Ags to CD8 T cells [8–10]. CD8α^−^ cDCs, another major population in mouse spleen cDCs, have the selective ability to directly present extracellular Ags to CD4 T cells [8, 11]. Since tumor vaccine needs to induce tumor Ag specific CTL activation for the efficient tumor cell-killing activity, CD8α^+^ DC-mediated cross-presentation of tumor Ags is crucially important [1, 2]. Moreover, due to limited CTL activation in the absence of helper T (Th) cell responses, CD4 T cell activation induced by matured CD8α^−^ DCs is also required [12].

Some of naturally occurred polysaccharides, bacterial products, and cytokines are known as immunological adjuvants for inducing immune activation against Ags [9, 13–16]. Adjuvant functions of seaweed polysaccharides have shown to be promising in tumor immunotherapy by inducing targeted tumor immunity [9, 15]. Ascophyllan extracted from brown alga *Ascophyllum nodosum* (*A. nodosum*) is a sulfated polysaccharide [17]. Previous studies showed that ascophyllan promotes activation of macrophages, NK cells and spleen DCs [17–19]. However, the effect of ascophyllan on immune cell activation in the *in vivo* tumor environment has not been investigated. Moreover, the possible function of ascophyllan as an adjuvant for tumor vaccines also has not been evaluated. The present study is undertaken to test whether *in vivo* administration of ascophyllan on B16 tumor-bearing mice can induce the activation of spleen DCs and the consequent activation and proliferation of Ag-specific T cells, to exert anti-tumor effect. In addition, we also investigated whether ascophyllan can function as an immunogenic adjuvant for the treatment and prevention of B16 melanoma in a mouse model.

## RESULTS

### Ascophyllan induces DC activation in the tumor environment

**Figure 1:**
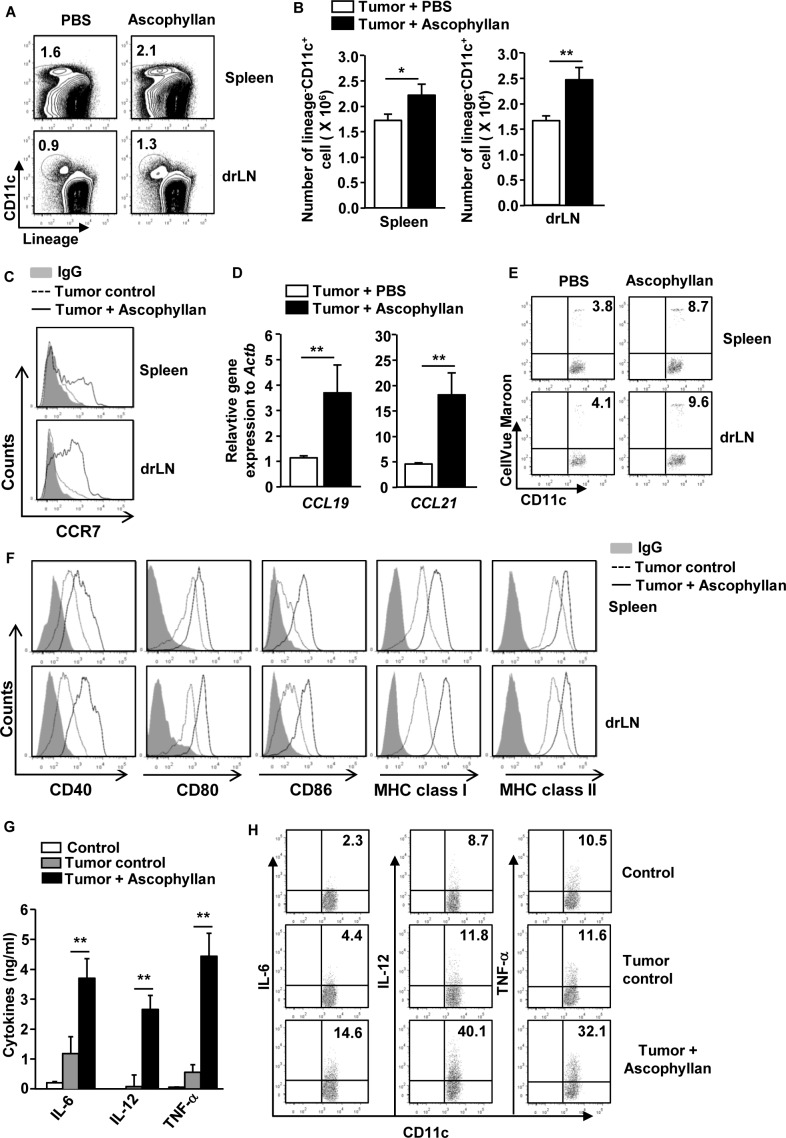
*In vivo* administration of ascophyllan induces maturation of DCs in the tumor-bearing mice C57BL/6 mice were injected *subcutaneously* (*s.c.*) with 1 × 10^6^ B16 melanoma cells. On day 10 after tumor cell injection, once tumors were well established, the mice were treated with 50 mg/kg ascophyllan for 24 hours and spleen and tumor draining lymph node (drLN) were harvested. **A.** Percentages of lineage^−^CD11c^+^ DCs in spleen and tumor drLN were analyzed on a flow cytometry. Lineage markers included CD3, Thy1.1, B220, Gr-1, CD49b and TER-119. **B.** Mean of absolute numbers of lineage^−^CD11c^+^ cells within live cells in spleen (left panel) and tumor drLN (right panel) are shown. **C.** Expression levels of CCR7 in gated lineage^−^CD11c^+^ cells from spleen and tumor drLN. **D.** Real-time PCR analysis of gene expression, presented relative to that of β-actin, in splenocytes. **E.** Isolated DCs from naïve mice were labeled with CellVeu Maroon and transferred into tumor-bearing mice. One hour after the DC transfer, the mice were treated with ascophyllan for 24 hours. Migration of CellVeu Maroon-labeled DCs was determined by flow cytometry. **F.** Flow cytometric analysis of co-stimulatory molecules and MHC class I and II in gated lineage^−^CD11c^+^ cells from spleen and tumor drLN. **G.** IL-6, IL-12p70 and TNF-α levels in sera are shown. **H.** Intracellular IL-6, IL-12 and TNF-α production in spleen DCs. All data are representative of or the average of analyses of 6 independent samples (2 mice per experiment, total 3 independent experiments). *p < 0.05, **p < 0.01.

Our previous study has shown that ascophyllan induces spleen DC maturation [19]. In this study, we assessed whether ascophyllan can also induce maturation of DCs in tumor-bearing mice. C57BL/6 mice were injected subcutaneously (*s.c.*) with 1 × 10^6^ B16 melanoma cells. Once tumors were well established, we intravenously (*i.v.*) injected with 50 mg/kg ascophyllan to these tumor-bearing mice and analyzed the activation of DCs in spleen and tumor draining lymph node (drLN) 24 hours later. Ascophyllan treatment led to marked increases in the proportion and number of DCs in spleen and tumor drLN (Figure 1A and 1B). C-C chemokine receptor 7 (CCR7) expression levels, the marker of migrating DCs [20], in spleen DCs and tumor drLN and its ligands C-C motif ligand 19 (CCL19) and CCL21 in spleen were substantially increased by ascophyllan (Figure 1C and 1D). To further determine whether ascophyllan can promote DC migration, we transferred CellVue Maroon-labeled DCs to tumor-bearing mice and treated them with ascophyllan one hour after transfer. As shown in Figure 1E, ascophyllan treatment promoted greater DC migration to spleen and tumor drLN compared to null-treated mice. Furthermore, the expression levels of co-stimulatory molecules and MHC class I and II on spleen and tumor drLN DCs were substantially up-regulated by ascophyllan (Figure 1F).

It is well known that matured DCs secrete pro-inflammatory cytokines including IL-6, IL-12 and TNF-α [2]. Therefore, we next examined whether ascophyllan can promote production of pro-inflammatory cytokines by DCs in tumor-bearing mice. We analyzed serum cytokine levels after 24 hours of ascophyllan treatment in tumor-bearing mice. As shown in Figure 1G, the levels of IL-6, IL-12p70 and TNF-α were substantially increased compared to PBS-treated tumor-bearing mice. To determine whether these cytokines were produced by ascophyllan-stimulated DCs, we analyzed intracellular cytokines production in spleen DCs. As shown in Figure 1H, ascophyllan treatment led to marked increases in the percentage of IL-6-, IL-12- and TNF-α-producing DCs in spleen of tumor-bearing mice. Thus, these data indicate that ascophyllan can induce migration of DCs to spleen and tumor drLN and promote activation of DCs in tumor-bearing mice.

### Ascophyllan promotes Th1 and Tc1 responses in the tumor-bearing mice

**Figure 2:**
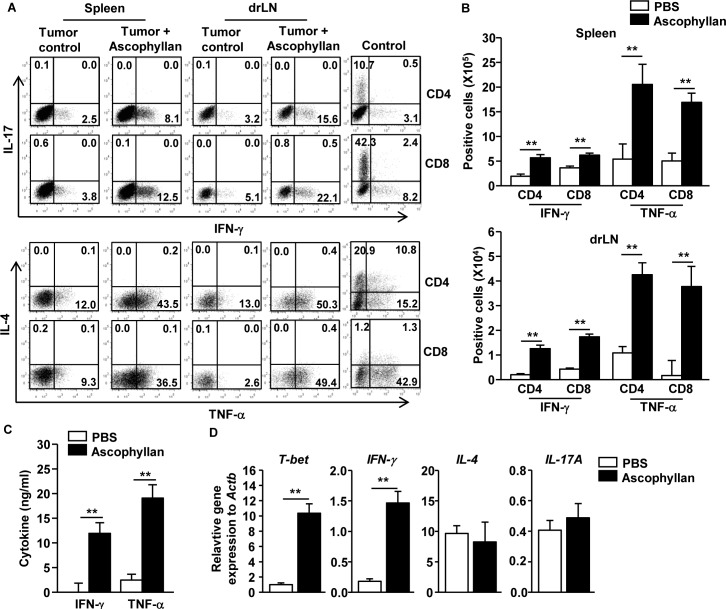
Ascophyllan promotes Th1 and Tc1 immune responses in the tumor-bearing mice On day 10 after B16 cell injection, once tumors were well established, the mice were injected *i.v.* with 50 mg/kg ascophyllan and 3 days later, injected again with same amount of ascophyllan for further 3 days. **A.** Percentage of IFN-γ^+^, IL-17^+^, TNF-α^+^, and IL-4^+^ in CD4^+^ and CD8^+^ T cells within spleen and tumor drLN. Control plots show IL-4 and IL-17 staining from CD4 and CD8 T cells on *in vitro* stimulated splenocytes. **B.** Mean of absolute numbers of IFN-γ^+^ and TNF-α^+^ cells within live cells in spleen (upper panel) and tumor drLN (lower panel) are shown. **C.** IFN-γ and TNF-α levels in sera are shown. **D.** Real-time PCR analysis of gene expression, presented relative to that of β-actin, in splenocytes stimulated with ascophyllan for 24 hours. All data are representative of or the average of analyses of 6 independent samples (2 mice per experiment, total 3 independent experiments). **p < 0.01.

Since ascophyllan can induce DC activation in tumor-bearing mice, we assessed whether ascophyllan-induced DC activation can promote T cell immune responses in tumor-bearing mice. Once B16 melanoma were established in mice, the mice received *i.v.* injection of 50 mg/kg ascophyllan twice, 3 days apart, and were analyzed 3 days after the second injection. Treatment of ascophyllan led to substantial increases in the proportions of IFN-γ- and TNF-α-producing CD4 and CD8 T cells in the spleen and tumor drLN, whereas IL-4- or IL-17-producing CD4 and CD8 T cells were not increased by ascophyllan treatment (Figure 2A). Consistent with flow cytometry data, the numbers of IFN-γ- and TNF-α-producing CD4 and CD8 T cells in spleen and tumor drLN were significantly increased by ascophyllan treatment (Figure 2B). Moreover, serum levels of IFN-γ and TNF-α were markedly elevated by ascophyllan treatment in the tumor-bearing mice (Figure 2C). Furthermore, mRNA levels of T-bet, the critical transcription factor for Th1 and Tc1 cells, and IFN-γ in splenocyte were also substantially increased by ascophyllan treatment, whereas IL-4 and IL-17A mRNA levels were not changed by ascophyllan (Figure 2D). Thus, these data suggest that ascophyllan treatment promotes Th1 and Tc1 responses in tumor-bearing mice.

### Ascophyllan enhances Ag presentation and Ag specific T cell proliferation in tumor-bearing mice

**Figure 3:**
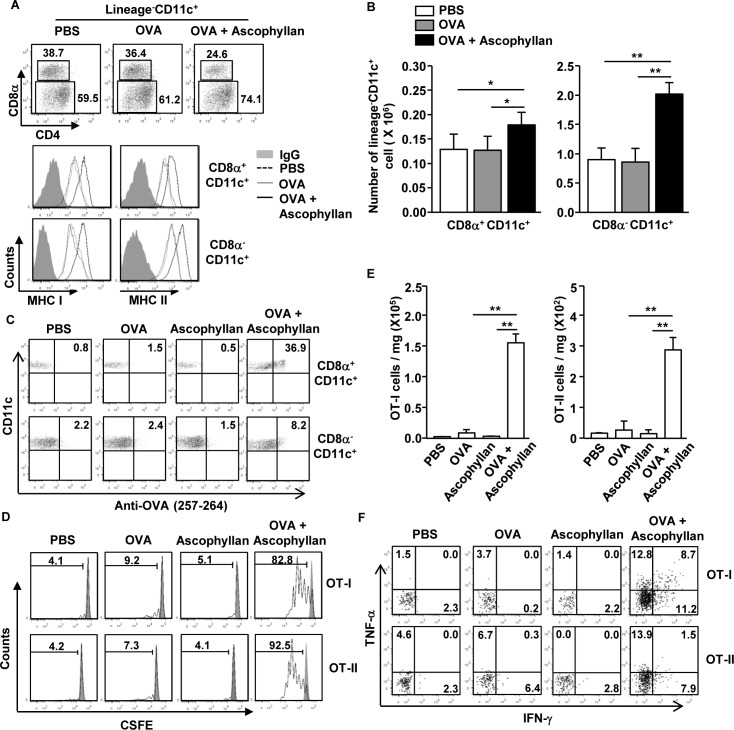
Ascophyllan promotes Ag-specific CD4 and CD8 T cell proliferation in the tumor-bearing mice B16 tumor-bearing C57BL/6 mice were injected with PBS, OVA or combination of ascophyllan and OVA for 24 hours. **A.** Percentage of lineage^−^CD11c^+^CD8α^+^ and lineage^−^CD11c^+^CD8α^−^ cDCs in spleen was analyzed on a flow cytometry (upper panel). Expression levels of MHC class I and II in the CD11c^+^CD8α^+^ and CD11c^+^CD8α^−^ cDCs are shown (lower panel). **B.** Mean of absolute number of CD11c^+^CD8α^+^ and CD11c^+^CD8α^−^ cDCs within live cells in spleen. **C.** Presentation of OVA peptide in the CD11c^+^CD8α^+^ and CD11c^+^CD8α^−^ cDCs was analyzed by Flow cytometry. **D.** Purified CD8 T cells from OT-I or CD4 T cells from OT-II mice were labeled with CFSE and transferred into B16 tumor-bearing CD45.1 congenic mice, and 24 hours later, mice were injected with PBS, OVA or combination of ascophyllan and OVA. On day 3 after ascophyllan and OVA injection, splenocytes from these mice were stained for CD45.2 to identify the donor OT-I or OT-II cells, and the proliferation of these cells was determined by CFSE dilution. **E.** Mean of absolute numbers of OT-I (left panel) and OT-II (right panel) cells in the tumor are shown. **F.** Percentage of IFN-γ^+^ and TNF-α^+^ cells in tumor-infiltrated OT-I and OT-II cells. All data are representative of or the average of analyses of 6 independent samples (2 mice per experiment, total 3 independent experiments). **p < 0.01, *p < 0.05.

Different subsets of DCs show different specialized functions for Ag presentation [10]. To determine whether ascophyllan can promote Ag-presentation or cross presentation in tumor-bearing mice, we first injected with PBS, ovalbumin (OVA) or a combination of ascophyllan and OVA to the tumor-bearing mice. After 24 hours, expression levels of MHC class I and II were measured on spleen CD8α^+^ and CD8α^−^ cDCs. The combination of ascophyllan and OVA treatment promoted dramatic up-regulation of MHC class I and II expression on both CD8α^+^ and CD8α^−^ cDCs compared to those in mice treated with OVA alone (Figure 3A). Moreover, the numbers of CD8α^+^ and CD8α^−^ cDCs in the spleen were significantly increased by the combination of ascophyllan and OVA (Figure 3B). Furthermore, the combination of ascophyllan and OVA-treated CD8α^+^ and CD8α^−^ cDCs resulted in a much higher percentage of OVA peptide presentation than treatment with OVA or ascophyllan alone (Figure 3C). Next, to determine Ag specific T cell proliferation, we transferred CFSE-labeled OT-I and OT-II cells into tumor-bearing CD45.1 congenic mice and 24 hours later, the mice were treated with PBS, OVA, ascophyllan or the combination of ascophyllan and OVA for 3 days. The combination of ascophyllan and OVA, but not OVA and ascophyllan alone, induced marked increases in the proliferation of OT-I and OT-II T cells in the spleen (Figure 3D). In addition, the numbers of tumor-infiltrating OT-I and OT-II cells were robustly increased in mice treated with the combination of ascophyllan and OVA (Figure 3E). Moreover, the intracellular production of IFN-γ and TNF-α in the tumor-infiltrating OT-I and OT-II cells in mice treated with the combination of ascophyllan and OVA were substantially increased compared to those treated with ascophyllan or OVA alone (Figure 3F). These data suggest that ascophyllan promotes Ag specific T cell responses and functions as an immunogenic adjuvant in tumor-bearing mice.

### OVA-immunization in conjunction with ascophyllan inhibits B16-OVA tumor cell growth

**Figure 4:**
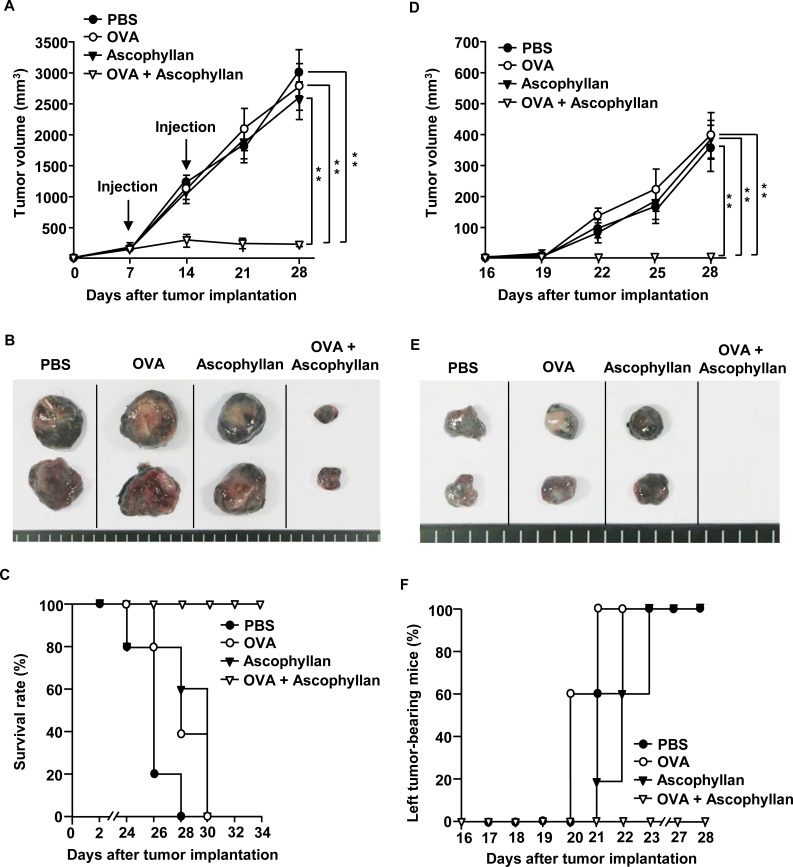
The combination treatment with ascophyllan and OVA inhibits B16-OVA tumor growth C57BL/6 mice were injected *s.c.* with 1 × 10^6^ B16-OVA cells on right side of mice. Once tumors were well established on day 7, mice received *i.v.* with PBS, OVA, ascophyllan or the combination of ascophyllan and OVA and 7 days later, treated with the same amount of OVA and ascophyllan again. **A.** B16-OVA tumor growth curves on right side of mice are shown. **B.** The size of tumor mass on day 28 after B16-OVA tumor cell challenge. **C.** Survival rate of mice is shown. **D.** On day 16 after B16-OVA cell challenge on right side, the mice were inoculated *s.c.* with 1 × 10^6^ B16-OVA cells on left side. Tumor growth on left side of mice is shown. **E.** The size of tumor mass on left side on day 28 after the initial tumor cell challenge on right side. **F.** Percentage of tumor-bearing mice on left side is shown. All data are representative of or the average of analyses of 5 independent samples (2 or 3 mice per experiment, total 2 independent experiments). **, statistically significant values, defined as *P < 0.01* and determined with paired Student's *t* test, compared with corresponding groups.

As ascophyllan promoted DC and Ag specific T cell activation in tumor-bearing mice, we hypothesized that ascophyllan may be used as an effective adjuvant in tumor immunotherapy. We first evaluated the effect of ascophyllan as an adjuvant for a therapeutic vaccine in tumor-bearing mice. C57BL/6 mice were inoculated *s.c.* with 1 × 10^6^ B16-OVA melanoma cells on the right side. After 7 days, once tumors were well established, mice were treated *i.v.* with PBS, OVA, ascophyllan or the combination of ascophyllan and OVA, and 7 days later, treated again with the same reagents. On day 16 after the initial tumor cell inoculation, the mice were inoculated again with the same numbers of B16-OVA cells on the left side. Treatment with the combination of ascophyllan and OVA dramatically inhibited the growth of B16-OVA tumors on the right side (Figure 4A). As shown in Figure 4B, mice treated with the combination of ascophyllan and OVA showed substantially smaller tumor mass on day 28 compared to those treated with OVA or ascophyllan alone. Moreover, mice treated with the combination of ascophyllan and OVA survived much longer than those treated with PBS, OVA or ascophyllan alone (Figure 4C). Furthermore, in mice treated with OVA or ascophyllan alone, the tumor mass on the left side resulting from the second tumor challenge were detected on day 28 after the initial tumor challenge on the right side, whereas mice treated with the combination of ascophyllan and OVA were completely protected from the second tumor challenge (Figure 4D, 4E and 4F), indicating that the combination of ascophyllan and OVA induced the formation of long-term memory immune responses against OVA Ag. These data suggest that ascophyllan can function as a therapeutic vaccine adjuvant.

### Ascophyllan functions as an adjuvant to enhance OVA-specific immune responses *in vivo*

**Figure 5:**
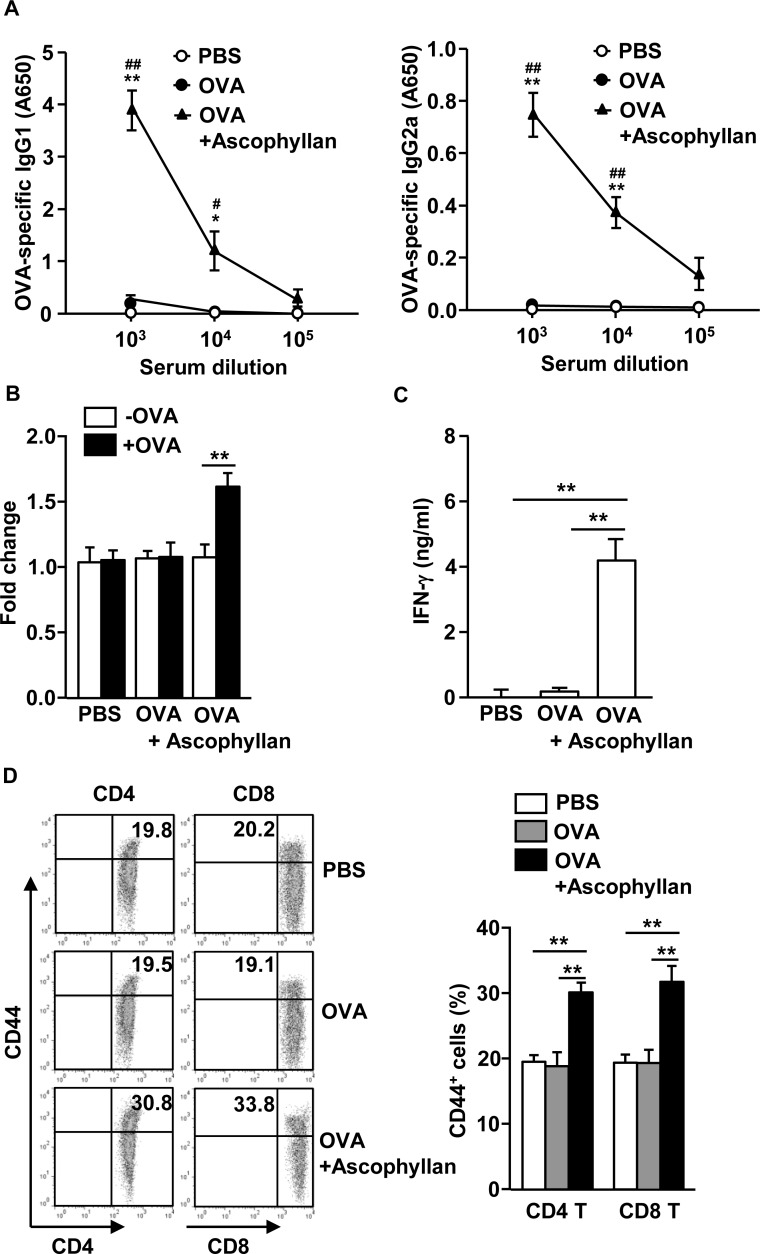
Ascophyllan provides an adjuvant effect on OVA-induced B and T cell responses C57BL/6 mice were immunized *i.v.* with PBS, OVA or the combination of OVA and ascophyllan on day 0, 15 and 30. **A.** On day 35, serum OVA-specific IgG1 (left panel) and IgG2a (right panel) concentrations were measured by ELISA. *P < 0.05, **P < 0.01 versus OVA group. ^#^*P < 0.05*, ^##^*P < 0.01 versus* PBS group. **B.** On day 35 after immunization, splenocytes were re-stimulated with or without OVA (50 μg/ml) for 4 days. Proliferation of splenocytes was measured. **C.** IFN-γ concentrations in the cultured supernatants of above splenocytes are shown. **D.** Expression of CD44 in CD4 or CD8 T cells was analyzed on a flow cytometry on day 35 (left panel). Mean percentages of CD44^+^ cells in CD4 or CD8 T cells are shown (right panel). All data are representative of 6 samples from 3 independent experiments. **p < 0.01.

Our observation that treatment with ascophyllan and OVA protected mice from second tumor challenge prompted us to examine whether OVA-immunization with ascophyllan promotes OVA-specific antibodies and T cell responses. C57BL/6 mice were injected *i.v.* with OVA alone or together with 50 mg/kg ascophyllan on day 0, 15 and 30. On day 35, we harvested serum and splenocyte for further analysis. OVA-immunization with ascophyllan induced significant increases in the OVA-specific immunoglobulin G1 (IgG1) and IgG2a levels in the serum compared to PBS treatment, whereas immunization with OVA alone did not have such effect (Figure 5A). On day 35, we also re-stimulated splenocytes with OVA *in vitro* for 4 days, and measured the T cell responses against OVA. Splenocytes from mice immunized with the combination of ascophyllan and OVA showed substantially greater cell proliferation and IFN-γ production than those from mice immunized with OVA alone (Figure 5B and 5C). Moreover, expression levels of CD44, a critical surface marker of effector/memory T cells, were significantly higher in spleen CD4 and CD8 T cells from mice immunized with the combination of ascophyllan and OVA than those from mice treated with PBS or OVA alone (Figure 5D). These data suggest that ascophyllan functions as an adjuvant to enhance production of Ag specific B cell and T cell immune responses.

### OVA-immunization with ascophyllan protects mice from B16-OVA tumor challenge

**Figure 6:**
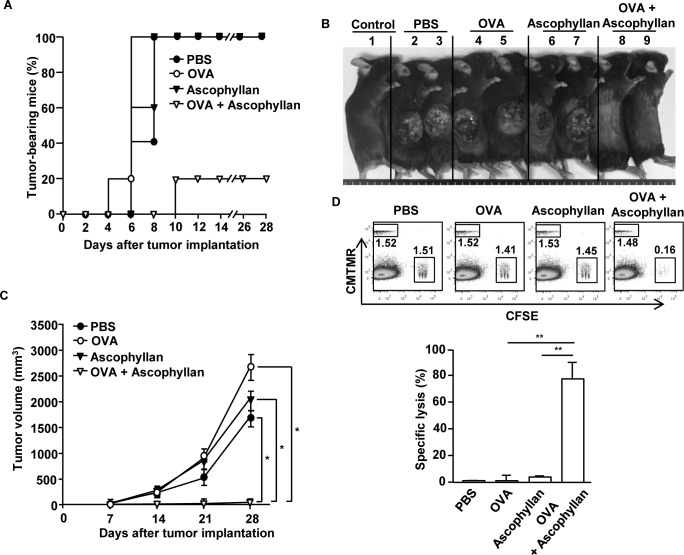
Immunization with ascophyllan and OVA protects mice from challenge with B16-OVA melanoma cells C57BL/6 mice were immunized with PBS, OVA, ascophyllan or the combination of ascophyllan and OVA on day 0, 15 and 30. On day 35 after immunization, the mice were challenged *s.c.* with 1 × 10^6^ B16-OVA melanoma cells. **A.** The percentages of tumor-bearing mice are shown. **B.** The pictures of tumor-bearing mice on day 28 are shown. **C.** Tumor growth curves are shown. All data are representative of or the average of analyses of 5 independent samples (2 or 3 mice per experiment, total 2 independent experiments). *, statistically significant values, defined as *P < 0.01* and determined with paired Student's t test, compared with corresponding groups. **D.** CTL activity was assessed *in vivo* at 35 days of immunization by adoptively transfer of splenocytes populations labeled with different concentrations of CFSE and loaded with SIINFEK, and also a control splenocyte population without peptide labeled with CMTMR. Dot plots show percentage of SIINFEK-loaded CFSE^+^ cells and non-peptide-loaded CMTMR^+^ cells (upper panel). Mean percentages of Ag-specific lysis (lower panel). Data are from analyses of 5 individual mice each group (2 or 3 mice per experiment, total 2 independent experiments). **p < 0.01.

Based on the observation that ascophyllan can function as an adjuvant for OVA-specific immune responses, we further examined whether this response can protect mice grafted with B16-OVA tumor cells. C57BL/6 mice were immunized *i.v.* with PBS, OVA, ascophyllan or the combination of ascophyllan and OVA on day 0, 15 and 30. On day 35, mice were inoculated *s.c.* with B16-OVA melanoma cells. Mice immunized with the combination of ascophyllan and OVA were almost completely protected from the B16-OVA tumor challenge (Figure 6A and 6B). Moreover, mice immunized with the combination of OVA and ascophyllan in mice showed markedly decreased tumor growth compared to those immunized with PBS, OVA or ascophyllan alone (Figure 6C). We next investigated the functional activity of CTLs in an *in vivo* cytotoxicity assay. On day 35 after the initial immunization, the immunized mice were injected *i.v.* with SIINFEKL-pulsed and CFSE-labeled splenocytes from C57BL/6 donor mice and measured for specific cell lysis by flow cytometry. Specific target cell lysis was 75% in mice immunized with the combination of ascophyllan and OVA, indicative of T cell memory induction (Figure 6D). In contrast, mice immunized with OVA or ascophyllan alone showed no significant killing of OVA-pulsed splenocytes (Figure 6D). Collectively, these data suggest that ascophyllan promotes the formation of long-term, tumor Ag specific immune responses *in vivo* and protects mice against the tumor challenge.

### OVA immunization in conjunction with ascophyllan inhibits B16-OVA melanoma metastasis

**Figure 7:**
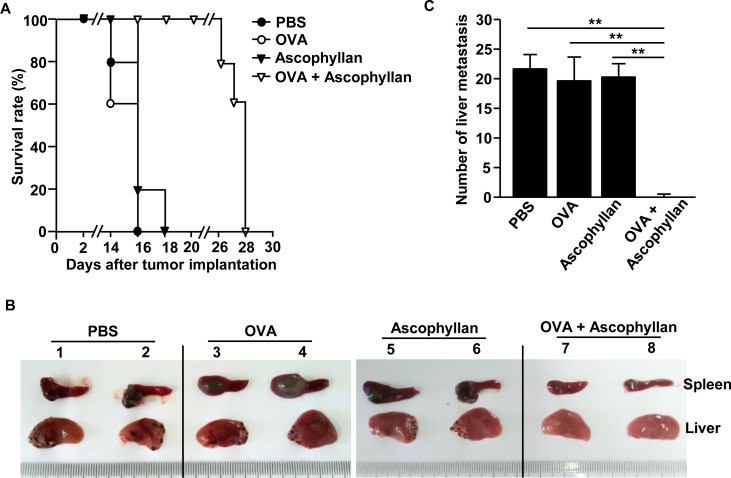
Treatment with ascophyllan and OVA prevents B16-OVA tumor cell metastasis into liver C57BL/6 mice were treated *i.v.* with PBS, 50 μg OVA, 50 mg/kg ascophyllan or the combination of ascophyllan and OVA. On day 3 after the injection, the mice were inoculated *intrasplenically* (*i.s*.) with B16-OVA melanoma cells. On day 3 after B16-OVA cell challenge, mice received same amount of ascophyllan and OVA treatment again. **A.** Survival rates of mice are shown. **B.** The size of tumor mass in spleen and metastasis of B16-OVA cells into the liver on day 10 after tumor injection are shown. **C.** Mean of absolute number of B16-OVA metastasis into the liver. All data are representative of or the average of analyses of 5 independent samples (2 or 3 mice per experiment, total 2 independent experiments). **p < 0.01.

We next examined whether treatment with the combination of ascophyllan and OVA can inhibit tumor metastasis in mice. Mice were injected *i.v.* with PBS, 50 μg OVA, 50 mg/kg ascophyllan or the combination of ascophyllan and OVA, and 3 days later, inoculated *intrasplenically* (*i.s*.) with 0.5 × 10^6^ B16-OVA melanoma cells. On day 3 after the tumor cell injection, the mice received the same amount of ascophyllan and OVA treatment again. Mice treated with PBS, OVA or ascophyllan alone started dying from day 14 and all the mice died within 18 days after the inoculation of B16-OVA cells. In contrast, mice treated with the combination of ascophyllan and OVA started dying from day 26 and all the mice died with 28 days after B16-OVA challenge (Figure 7A). Moreover, the size of tumor mass in the spleen at day 10 after injection of B16-OVA cells was much smaller in mice that treated with the combination of ascophyllan and OVA compared to those treated with OVA or ascophyllan alone (Figure 7B). Furthermore, the mice treated with the combination of ascophyllan and OVA showed much lower degree of tumor cell invasion and metastasis in the liver compared to all the other treatment groups, in which metastatic tumor cells were observed in the liver of all the mice (Figure 7B and 7C). Thus, these data suggest that the combination of ascophyllan and OVA protects against liver invasion and metastasis of B16-OVA melanoma cells.

## DISCUSSION

Adjuvant for cancer vaccine should boost cell-mediated immune responses in order to effectively prevent cancer development [21, 22]. Since cancer Ag is unable to efficiently promote Ag-specific immune responses, effective adjuvant for cancer vaccine is required [3, 4]. In this study, we found a novel promising candidate of vaccine adjuvant that may be applicable for human use in cancer therapy and prevention. We found that ascophyllan treatment promoted soluble OVA presentation by both CD8α^+^ and CD8α^−^ cDCs, which in turn enhanced OVA specific CD4 and CD8 T cell proliferation. In addition, the combination of ascophyllan and OVA promoted tumor-infiltration of OVA specific T cell that potently produced IFN-γ and TNF-α. Moreover, Ascophyllan promoted OVA specific memory T cell differentiation and CTL activation. Finally, treatment with the combination of ascophyllan and OVA showed therapeutic and preventive effects on tumor in mice challenged with B16-OVA melanoma cells. Taken together with our previous study that ascophyllan administration in mice did not cause tissue damage [19], these findings suggest that ascophyllan may be potentially utilized as a cancer adjuvant for human uses.

Activation of DCs by stimuli induces the process of DC maturation, which is defined by increased expression of co-stimulatory molecules and production of pro-inflammatory cytokines [2, 6]. Our study showed that ascophyllan induces maturation of spleen and tumor drLN DCs in the tumor environment *in vivo*, and importantly, that ascophyllan induces maturation of both CD8α^+^ and CD8α^−^ cDCs. CD8α^+^ cDCs are specialized in cross presentation of Ags *via* MHC class I to CTLs, and therefore enhancing CD8α^+^ cDC activation for tumor vaccine has been demonstrated as a new promising strategy in vaccine development [22–24]. In line with this, ascophyllan promoted activation of CD8α^+^ cDCs and OVA specific CTL proliferation and activation, which consequently induced specific killing of OVA-pulsed splenocytes. Therefore, these data suggest that ascophyllan-induced maturation of CD8α^+^ cDCs may promote Ag specific CTL activation. Moreover, ascophyllan also induced activation of OVA specific CD4 T cells. Since matured CD8α^−^ cDCs are specialized to promote activation and proliferation of CD4 T cells [8, 11], ascophyllan-induced CD4 T cell activation may be promoted by ascophyllan-activated CD8α^−^ cDCs. Hence, these data suggest that ascophyllan may have the ability to enhance not only cross-presentation of Ags by CD8α^+^ cDCs but also direct presentation of Ags by CD8α^−^ cDCs.

Therapeutic vaccines for cancer must circumvent tumor microenvironment-induced immune suppression that limits the activation of DCs and T cells [5]. Thus, in the tumor microenvironment, effective therapeutic vaccines require adjuvants as components to overcome immune-suppression and enhance Ag specific immune activation [21, 22, 25]. In this study, we evaluated the effect of ascophyllan in tumor-bearing mice, and found that ascophyllan induced DC maturation and T cell activation in the spleen and tumor drLN, and promoted OVA-specific T cell proliferation and migration into the tumor, which consequently resulted in significant therapeutic effect against B16-OVA melanoma. Thus, these data suggest that ascophyllan can be used as a therapeutic vaccine adjuvant.

Interestingly, ascophyllan treatment does not induce any change in the number of spleen DCs in the tumor free mice [19], but in this study, it induced an increase in the number of spleen and tumor drLN DCs in the tumor-bearing mice. Since DCs are not able to proliferate, this increase may be a result of augmented DC migration to the spleen and tumor drLNs induced by ascophyllan. It has been shown that in tumor microenvironment, inhibited chemokine expression impairs DC migration to the spleen [26]. Moreover, ascophyllan treatment induced up-regulation of CCR7 in DCs and its ligands CCL19 and CCL21 in spleen in tumor-bearing mice. These data suggest that ascophyllan can enhance chemokine expression and promote DC migration to spleen and tumor drLNs in tumor-bearing conditions but may not promote chemokine expression in the naïve condition.

Since natural products have relatively low or tolerable toxicities, they are recognized as promising candidates for immunomodulatory agents [27]. Some marine-derived sulfated polysaccharides have been shown to possess strong immune-activating effect [9, 15, 28, 29]. In this study, we also found that ascophyllan can function as an adjuvant to enhance Ag-specific immune responses and anti-tumor and anti-metastasis activities. Our previous study demonstrated that ascophyllan-induced *in vivo* DC activation is stronger than those induced by fucoidan, a marine-derived polysaccharide with well-defined effect on promoting DC maturation [19], suggesting that ascophyllan may be a more effective adjuvant candidate than fucoidan for the development of novel therapeutic and protection vaccine strategies.

In conclusion, our results provide several lines of evidence that the ascophyllan is a novel adjuvant that can induce immune activation including DC maturation, Th1 immune responses, CTL activation, Ag-specific antibody production and memory T cell generation. The adjuvant function of ascophyllan will be potentially useful for developing tumor vaccines for human uses.

## MATERIALS AND METHODS

### Mice and cell lines

C57BL/6 mice (6 weeks old), OT-I and OT-II TCR transgenic mice and C57BL/6-Ly5.1 (CD45.1) congenic mice were obtained from Shanghai Public Health Clinical Center, and kept under pathogen-free conditions. The mice were maintained in a room with controlled temperature (20-22°C), humidity (50-60%) and light (12 h: 12 h) with free access to standard rodent chow and water. All experiments were carried out under the guidelines of the Institutional Animal Care and Use committee at the Shanghai Public Health Clinical Center. The protocol was approved by the committee on the Ethics of Animal Experiments of the Shanghai Public Health Clinical Center (Mouse Protocol Number: SYXK-2010-0098). Mice were sacrificed by CO_2_ inhalation euthanasia, and all efforts were made to minimize suffering. The murine melanoma cell line B16F10 (ATCC, CRL-6475) expressing OVA (B16-OVA) was cultured in RPMI 1640 (Sigma Aldrich, 10% FBS, 2 mM glutamine, 1 M HEPES, 100 μg/ml streptomycin and 100 U/ml penicillin and 2 mM 2-mercaptoethanol). All cell lines were cultured at 37°C in a humidified atmosphere of 5% CO_2_ and air.

### Chemicals and cytokines

Ascophyllan was prepared from the powdered *A. nodosum* as described previously [17, 30]. Ascophyllan solution was passed through an endotoxin-removal column (Detoxi-gel: Thermo Fisher Scientific), and subsequently filtered through an endotoxin-removal filter (Zetapor Dispo: Wako). The endotoxin levels in purified ascophyllan were evaluated using a Limulus amebocyte lysate (LAL) assay kit (Lonza). The H2-K^b^ restricted peptide OVA257-264 (SIINFEKL) was purchased from Chinapeptides (China).

### Antibodies

Isotype control antibodies (Abs) (IgG1, IgG2a or IgG2b), CD11c (HL3), CD4 (GK1.5), CD8α (YTS169.4), CD40 (3/23), CD80 (16-10A1), CD86 (GL-1), CCR7 (4B12), anti-IL-4 (11B11), anti-IL-6 (MP5-20F3) and anti-IL-12/23p40 (C17.8) were from BioLegend; anti-MHC class I (AF6-88.5.3), anti-MHC class II (M5/114.15.2), anti-IFN-γ (XMG1.2), anti-IL-17 (TCC11-18H10.1) and anti-TNF-α (MP6-XT22) were from eBioscience.

### Flow cytometry analysis

Cells were washed with phosphate buffered saline (PBS) containing 0.5% BSA, pre-incubated for 15 min with unlabeled isotype control Abs, and then labeled with fluorescence-conjugated Abs by incubation on ice for 30 min followed by washing with PBS. Cells were analyzed on a FACS Aria II (Becton Dickinson) and FlowJo 8.6 software (Tree Star). Cellular debris was excluded from the analysis by forward- and side-scatter gating. Dead cells were further excluded by 7 aminoactinomycin D (7AAD) (BioLegend) staining and gating on the 7AAD-negative population. As a control for nonspecific staining, isotype-matched irrelevant mAbs were used.

### DC analysis

Spleen and tumor drLN DCs were analyzed as described elsewhere [9, 19]. Briefly, the tissues were cut into small fragments and digested, with 2% fetal bovine serum (FBS) containing collagenase for 20 min at room temperature. Cells from the digest were centrifuged to a pellet, and the pellet was re-suspended in 5 mL of a 1.077 histopaque (Sigma-Aldrich). Additional histopaque was layered below and EDTA-FBS was layered above the cell suspension, which was then centrifuged at 1700g for 10 min. The light density fraction (< 1.077 g/cm^3^) was collected and incubated for 30 min with the following FITC-conjugated monoclonal antibodies (mAbs): anti-CD3 (17A2), anti-Thy1.1 (OX-7), anti-B220 (RA3-6B2), anti-Gr1 (RB68C5), anti-CD49b (DX5) and anti-TER-119 (TER-119). The lineage^−^CD11c^+^ cells were defined as cDCs, which were further divided into CD8α^+^ and CD8α^−^ cDCs. Analysis was carried out on a FACS Aria II (Becton Dickinson).

### DC migration assay

CD11c^+^ spleen DCs were isolated from naïve C57BL/6 mice by biotin-conjugated anti-CD11c Abs (Miltenyibiotec), and the cells were labeled with the CellVue Maroon cell labeling kit (eBioscience). The labeled cells were then transferred to tumor-bearing mice, and the mice received either ascophyllan or no ascophyllan 1 hour after DC transfer. Spleen and tumor drLN migrated cells were analyzed from lineage^−^CD11c^+^ cells by flow cytometry.

### *Ex vivo* T cell stimulation and intracellular cytokine staining

As described in detail previously [31], single cell suspension prepared from spleen and tumor drLN were stimulated *in vitro* for 4 hours with phorbol 12-myristate 13-acetate (50 ng/ml) and ionomycin (1 μM; both from Calbiochem), with the addition of monensin solution (Biolegend) during the final 2 hours. For positive control, splenocytes (2 × 10^6^/ml) from C57BL/6 mice were stimulated with soluble anti-CD3 (1 μg/ml) and anti-CD28 (1 μg/ml) for 3 days. IL-4 (Peprotech) or IL-6 (Peprotech) was added in the culture. Cells were then measured intracellular IL-4 and IL-17 production, respectively. For intracellular cytokine staining, cells were stained for surface molecules first, then fixed and permeabilized with Cytofix/Cytoperm buffer (eBioscience) and subsequently incubated with anti-cytokine antibodies in Perm/Wash buffer (eBioscience) for 30 min. Control staining with isotype control IgGs was performed in all experiments.

### ELISA

IL-6, IL-12p70, IFN-γ and TNF-α concentrations in the sera were measured in triplicate using standard ELISA kits (Biolegend).

### Real-time PCR

Total RNA was reverse-transcribed into cDNA using Oligo (dT) and M-MLV reverse transcriptase (Promega). The cDNA was subjected to real-time PCR amplification (Qiagen) for 40 cycles with annealing and extension temperature at 60°C, on a LightCycler 480 Real-Time PCR System (Roche). Primer sequences are: mouse β-actin forward, 5′-TGGATGACGATATCGCTGCG-3′; reverse, 5′-AGGGTCAGGATACCTCTCTT-3′, CCL19 forward, 5′-CTGCCTCAGATTATCTGCCAT-3′; reverse, 5′-AGGTAGCGGAAGGCTTTCAC-3′, CCL21a forward, 5′-ATCCCGGCAATCCTGTTCTC-3′; reverse, 5′-GGTTCTGCACCCAGCCTTC-3′, T-bet forward, 5′-CAACAACCCCTTTGCCAAAG-3′; reverse, 5′-TCCCCCAAGCATTGACAGT-3′, IFN-γ forward, 5′-GGATGCATTCATGAGTATTGC-3′; reverse, 5′-CTTTTCCGCTTCCTGAGG-3′, IL-4 forward, 5′-ACAGGAGAAGGGACGCCAT-3′; reverse 5′-GAAGCCCTACAGACGAGCTCA-3′, IL-17A forward, 5′-GCGCAAAAGTGAGCTCCAGA-3′; reverse 5′-ACAGAGGGATATCTATCAGGG-3′.

### Mouse immunization

C57BL/6 mice were immunized *i.v.* with PBS alone, 2.5 mg/kg of OVA in PBS, 50 mg/kg ascophyllan or OVA mixed with ascophyllan in PBS on days 0, 15 and 30. On day 35, mice were sacrificed, sera were collected, and splenocytes were harvested for further analysis. Some parts of mice were challenged with B16-OVA melanoma tumor.

### OVA-specific antibody analysis

As described in detail previously [9], 96-well plates were coated with OVA (10 μg/ml) and blocked with 1% bovine serum albumin (BSA). Serum samples were diluted and added to each well, followed by incubation with biotin-conjugated anti-mouse IgG1 and IgG2a (Biolegend) and streptavidin-conjugated HRP. The reaction was developed by TMB substrate (Sigma), and A_650_ was measured using a plate reader.

### OT-I and OT-II T cell proliferation

CD4 T cells from OT-II mice or CD8 T cells from OT-I mice were isolated from spleens using CD4 T cell or CD8 T cell isolation kit (Miltenyi Biotec), respectively. The cells were suspended in PBS/0.1% BSA containing 10 μM CFSE (Invitrogen) and incubated for 10 min. CFSE-labeled cells (1 × 10^6^) were *i.v.* transferred into CD45.1 congenic mice, and 24 hours later, mice were injected with PBS alone, 2.5 mg/kg of OVA in PBS or combination of ascophyllan and OVA in PBS. At 72 hours after immunization, splenocytes were harvested and OT-I or OT-II T cell proliferation was determined by analyzing the CFSE fluorescence intensity through flow cytometry.

### *In vivo* cytotoxicity assay

Mice were injected *i.v.* with a mixture of splenocytes differentially labeled with CFSE (2, 20, or 200 nM) and loaded with 1, 10, or 100 nM SIINFEKL peptide, respectively, and spleen cells labeled with 10 mM CellTracker^TM^ Orange CMTMR (Life technologies) and not loaded with peptide. A total of 10 × 10^6^ cells per mouse were injected, consisting of a mixture containing each target cell population. Splenocytes were collected 24 hours after injection of target cells. Presence of viable target cells was determined using exclusion by 7-aminoactinomycin D. Percentage killing was calculated using the formula as described [32].

### Intrasplenic injection

C57BL/6 mice were anesthetized with a ketamine mixture (10 μL ketamine HCl, 7.6 μL xylazine, 2.4 μL acepromazine maleate, and 10 μL H_2_O) injected into the peritoneal cavity. B16-OVA melanoma cells (0.5 × 10^6^/50 μL) were injected into the spleen of the mice during open laparotomy for experiments.

### Statistical analysis

Results are expressed as the mean ± standard error of the mean (SEM). The statistical significance of differences between experimental groups was calculated using analysis of variance with a Bonferroni post-test or an unpaired Student's t-test. All *p-values <0.05* were considered significant.
